# Crowd Gathering Detection Method Based on Multi-Scale Feature Fusion and Convolutional Attention

**DOI:** 10.3390/s25216550

**Published:** 2025-10-24

**Authors:** Kamil Yasen, Juting Zhou, Nan Zhou, Ke Qin, Zhiguo Wang, Ye Li

**Affiliations:** 1 School of Computer Science and Engineering, University of Electronic Science and Technology of China, Chengdu 610054, China; merkamilyasen@163.com (K.Y.); 202321090208@std.uestc.edu.cn (J.Z.); zn_986@163.com (N.Z.); qinke@uestc.edu.cn (K.Q.); zgwang@uestc.edu.cn (Z.W.); 2 Kashi Institue of Electronics and Information Industry, Kashi 844099, China

**Keywords:** deep learning, crowd gathering detection, convolutional attention

## Abstract

With rapid urbanization and growing population inflows into metropolitan areas, crowd gatherings have become increasingly frequent and dense, posing significant challenges to public safety management. Although existing crowd gathering detection methods have achieved notable progress, they still face major limitations: most rely heavily on local texture or density features and lack the capacity to model contextual information, making them ineffective under severe occlusions and complex backgrounds. Additionally, fixed-scale feature extraction strategies struggle to adapt to crowd regions with varying densities and scales, and insufficient attention to densely populated areas hinders the capture of critical local features. To overcome these challenges, we propose a point-supervised framework named Multi-Scale Convolutional Attention Network (MSCANet). MSCANet adopts a context-aware architecture and integrates multi-scale feature extraction modules and convolutional attention mechanisms, enabling it to dynamically adapt to varying crowd densities while focusing on key regions. This enhances feature representation in complex scenes and improves detection performance. Extensive experiments on public datasets demonstrate that MSCANet achieves high counting accuracy and robustness, particularly in dense and occluded environments, showing strong potential for real-world deployment.

## 1. Introduction

With accelerating urbanization and continuous population growth, major cities are facing increasing population density and spatial congestion, particularly in popular areas during holidays. Such conditions heighten the risk of abnormal crowd gatherings, posing safety concerns and underscoring the need for accurate and timely crowd gathering detection models. Currently, density map-based methods [1] are widely adopted to estimate crowd gatherings through the integration of predicted density maps. However, for tasks that require fine-grained analysis, such as crowd localization [2,3], tracking [4], and activity recognition, count estimation is inadequate since precise individual locations are essential. To address these issues, some studies use point annotations to generate pseudo bounding boxes [5,6,7], while others estimate crowd distribution via single head points [8,9,10]. The former relies on Fast RCNN [11] but lacks anchor box information, limiting accuracy. The latter bypasses bounding box estimation and directly outputs head points, often requiring postprocessing. However, both methods face challenges in head localization under uneven crowd distributions. Furthermore, a general point-supervised gathering detection model applicable across datasets remains absent. To address these issues, we proposed an adaptive scale convolutional attention module and a multi-scale convolutional fusion module. Experiments demonstrate that our model performs robustly under heavy occlusion and generalizes well across multiple datasets. Our main contributions are as follows: (1) We proposed a scale-adaptive attention module that enables the model to handle large variations in crowd distribution during training. (2) We designed a multi-scale convolutional feature fusion module to improve head detection performance in heavily occluded scenes. (3) We merged two existing crowd gathering datasets to form a new benchmark, and conducted cross-dataset validation to demonstrate our model’s generalization ability.

## 2. Related Work

In crowd gathering detection, CNN has shifted focus from handcrafted features to deep learning, mainly in two directions: density map-based and point-supervised methods. This section reviews these approaches.

### 2.1. Crowd Gathering Detection Method Based on Density Maps

Some early works combined CNNs with deep models [12] and introduced multi-task learning frameworks [13] to estimate crowd density levels, enhancing gathering detection with additional semantic information. Later studies improved performance through optimized loss functions [14,15,16]. Recently, methods have focused on pixel-level density map regression [16,17,18] or classifying local counts into intervals [19,20,21] to reduce inconsistencies between density maps and actual counts, with some incorporating contextual information [22]. Despite their success, density map-based methods lack instance-level predictions and cannot accurately locate individuals, facing limitations highlighted in [23]. In contrast, our method outputs individual head coordinates, providing more precise and intuitive crowd understanding.

### 2.2. Crowd Gathering Detection Method Based on Point Supervision

Unlike density map-based methods, some methods like [5,6,24] are inspired by cutting-edge object detectors and attempt to predict bounding boxes for individual heads. However, the pseudo ground-truth boxes generated from weak point supervision are often error-prone, especially in congested regions. This not only hinders effective model training but also results in inaccurate box predictions. In contrast, other methods like [9] rely on point annotations to detect gatherings, but they often face challenges in crowded scenes, such as handling duplicate detections or separating individuals who are too close to each other, other methods like [25] perform poorly due to excessive suppression of candidate instances. Therefore, there is a need for methods that can simplify point-supervised crowd gathering detection. For example, P2PNet [10] addresses this issue by directly estimating individual positions. Building upon these approaches, point-supervised methods aim to overcome the limitations of both bounding box prediction and postprocessed point detection by bypassing intermediate representations altogether. They directly estimate both individual locations and overall counts from annotations. Recent studies have further enhanced this paradigm through pixel-level regression, count interval classification, and contextual modeling. Nevertheless, density map-based methods still suffer from inherent limitations, particularly their inability to provide instance-level predictions and precise localization [23]. In contrast, our method outputs individual head coordinates, offering a more accurate and intuitive representation of the crowd.

## 3. Our Work

### 3.1. Introduction to Basic Model

**Figure 1:**
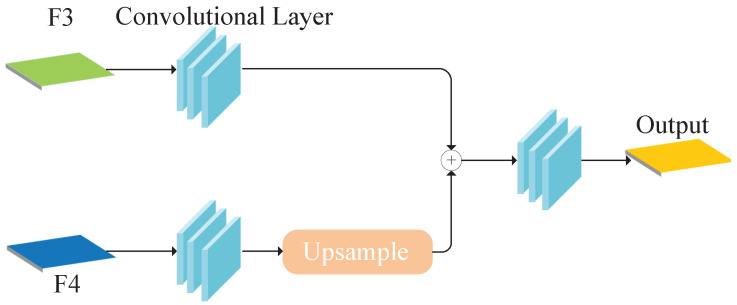
Feature pyramid module architecture.

P2PNet [10] is the first point-supervised crowd gathering detection model to achieve outstanding performance in this field. It achieves excellent results through a simple model architecture. First, the input image is processed through the hierarchical outputs of the VGG16 backbone to obtain multi-scale feature maps. These feature maps are then passed into a feature pyramid module, where higher-level maps are upsampled and fused with lower-level maps via convolutional layers to generate the final feature representation. The structure is illustrated in Figure 1, and the process is formulated as follows:

(1) $$F_{\mathrm{out}\;}={\mathrm{Conv}}_{3}\left({\mathrm{Conv}}_{1}\left(F_{3}\right)+\mathrm{Upsample}\left({\mathrm{Conv}}_{2}\left(F_{4}\right)\right)\right).$$

Let $F_{3}$ and $F_{4}$ denote the third and fourth feature maps from the VGG16 backbone. The fused features are fed into regression and localization branches and matched with ground truth points to generate the final output. While this architecture performs well on several datasets, it struggles in crowded and occluded scenes. To address these challenges, we propose MSCANet.

### 3.2. Our Model

**Figure 2:**
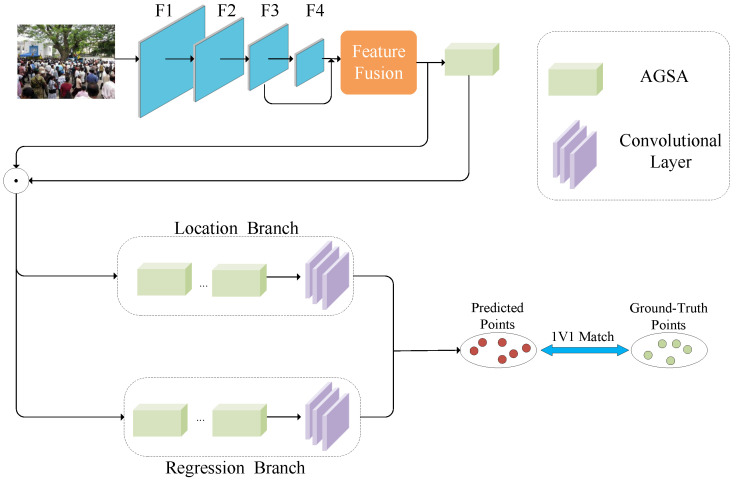
Overall architecture of our model.

As shown in Figure 2, our model includes five main components: feature extraction, feature fusion, attention enhancement, localization, and regression branches. The structure of each part is described below.

**Figure 3:**
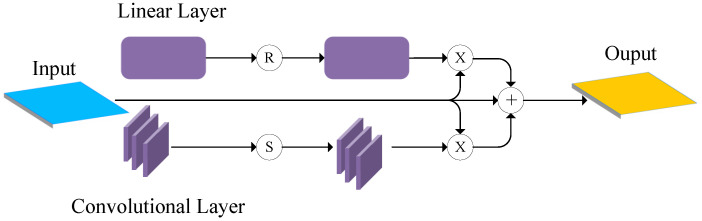
Attention enhancement module.

Our model uses the same VGG16-BN backbone and feature fusion module as P2PNet for initial feature extraction and fusion. Existing crowd counting datasets exhibit large variations in density, scale, and heavy occlusions. Current models often fail to effectively capture multi-scale features and handle occluded targets because of limited feature representation. To overcome these limitations, we propose an attention enhancement module that includes channel and spatial attention blocks, as shown in Figure 3 [26].

In the channel attention module, given an input feature map $X\in R^{B\times C\times H\times W}$, the feature map is first transformed from shape [B,C,H,W] to [B,H,W,C], and then flattened into tensor [B,−1,C] and represented as $X_{\mathrm{permute}}$. Then the tensor passes through a two-layer fully connected network with a hidden layer of size C/rate and an activation function Relu. The output is reshaped back to the original feature map size [B,H,W,C] as follows:

(2) $$X_{\mathrm{channel}\_\mathrm{out}}=W_{2}\cdot \mathrm{Relu}\left(W_{1}\cdot X_{\mathrm{permute}}+b_{1}\right)+b_{2}.$$

Finally, the channel attention weights are multiplied with the input feature map to produce a weighted feature map for further processing. The formula is as follows:

(3) $$X_{\mathrm{channel}\_\mathrm{out}}=X\cdot X_{\mathrm{channel}\_\mathrm{out}}.$$

The spatial attention module highlights important spatial positions using two convolutional layers. The first reduces channels from *C* to C/rate with batch normalization and Relu activation. The second restores the original channels with batch normalization, as follows:

(4) $$X_{\mathrm{spatial}\_\mathrm{out}}=\mathrm{BN}\left({\mathrm{Conv}}_{2}\left(\mathrm{Relu}\left(\mathrm{BN}\left({\mathrm{Conv}}_{1}\left(X\right)\right)\right)\right)\right).$$

The spatial attention output is activated by Sigmoid activation to range between 0 and 1, and then multiplied with the input feature map to obtain the final spatial output, as follows:

(5) $$X_{\mathrm{spatial}\_\mathrm{out}}=X\cdot \sigma \left(X_{\mathrm{spatial}\_\mathrm{out}}\right).$$

The final output of the attention enhancement module is the element-wise sum of the channel attention output, spatial attention output, and original input feature map, as follows:

(6) $$X_{out}=X+X_{\mathrm{channel}\_\mathrm{out}}+X_{\mathrm{spatial}\_\mathrm{out}}.$$

**Figure 4:**
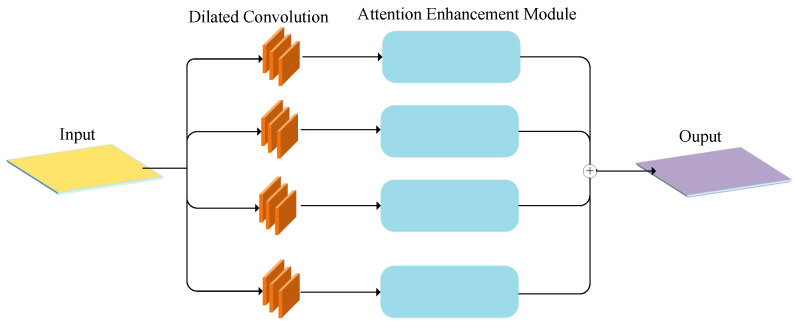
Attention guided scale adaptation module.

*X* is the original input, and $X_{\mathrm{channel}\_\mathrm{out}}$ and $X_{\mathrm{spatial}\_\mathrm{out}}$ are the outputs of channel and spatial attention. Combining them helps the network focus on important features in both channel and spatial dimensions, improving feature representation and model performance. To better handle occluded targets, we improved the model into the Attention Guided Scale Adaptation (AGSA) module with four parallel channels. Each channel has an expansion convolution (rates 1–4), followed by an attention enhancement module. These parallel pathways allow the model to capture multi-scale contextual information more effectively, improving feature selection. The attention enhancement module further refines the feature selection by suppressing irrelevant features and enhancing key ones, making the model more robust to scale variations and occlusions in the target features. The outputs are then summed element-wise for the final attention regression, enabling more robust handling of scale variations and occlusions in the target features, as shown in Figure 4.

The module output serves as a “soft switch”, performing element-wise multiplication with the fused multi-scale feature maps. By integrating convolutional attention into the multi-scale representation, it adaptively suppresses less informative features while emphasizing scale-specific and contextually critical ones. This joint design enhances the model’s ability to capture fine-grained spatial cues and scale variations within the context-aware architecture. The refined feature maps are then passed to the regression and localization branches. Each branch produces *M* candidate points, with M>N to ensure sufficient matching to the *N* ground-truth points. Both branches adopt the same structure, consisting of three convolutional attention enhancement modules followed by a convolutional layer. The localization branch outputs coordinates, while the regression branch provides confidence scores.

In the localization branch, predicting offsets works better than directly predicting exact positions due to convolution’s translational invariance. Therefore, we first choose fixed reference points $F=\left\{f_{i}|i\in \left\{1,2,\ldots ,M\right\}\right\}.$

Here, $f_{i}=\left(x_{i},y_{i}\right)$ is the fixed reference point coordinate, and the localization branch predicts its offset $\left(\Delta x^{c},\Delta y^{c}\right)$. All the fixed reference points are densely distributed across the patch. In our experiments, the number of those points is fixed to 1024. The predicted position of each candidate point is

(7) $$P^{c}=\left\{p_{i}^{c}|i\in \left\{1,2,\ldots ,M\right\}\right\}\;\mathrm{where}\;p_{i}^{c}=\left(x_{i}+\Delta x^{c},y_{i}+\Delta y^{c}\right).$$

The regression branch directly predicts confidence scores for *M* candidate points $S^{c}=\left\{s_{i}^{c}|i\in \left\{1,2,\ldots ,M\right\}\right\}$. Higher scores indicate a greater likelihood of being a final prediction. After obtaining positions and confidence scores, a one-to-one matching mechanism selects the predicted points. Matching weights $W(p_{i}^{c},{\hat{p}}_{j}^{c},s_{k}^{c})$ are calculated based on the distance and confidence between candidate and real points, as follows:

(8) $$W\left(p_{i}^{c},{\hat{p}}_{j},s_{i}^{c}\right)=\varepsilon {∥p_{i}^{c}-{\hat{p}}_{j}∥}_{2}-s_{i}^{c}.$$

Here, ${∥\cdot ∥}_{2}$ is the Euclidean distance, $p_{i}^{\epsilon }$ is the i-th candidate point, $s_{i}^{\epsilon }$ is its confidence score, ${\hat{p}}_{j}$ is the j-th ground-truth point, and ϵ is a loss balance weight set to 0.05. Finally, the Hungarian algorithm selects the final predicted points $P=\{{\hat{p}}_{j}|j\in \left\{1,2,\ldots ,N\right\}\}$ by minimizing the total matching weight.

### 3.3. Loss Function

The model predicts the input image and outputs coordinate points and confidence scores separately. These are matched one-to-one with ground-truth points. For the localization branch, the average Euclidean distance between predicted and true points is used as the training target. The formula is

(9) $$L_{\mathrm{loc}}=\frac{1}{N}\sum_{i=1}^{N}{∥p_{i}^{c}-{\hat{p}}_{j}^{c}∥}^{2}.$$

The classification branch uses cross-entropy loss to measure error, as follows:

(10) $$L_{\mathrm{cls}}=-\frac{1}{M}\left\{\sum_{I=1}^{N}\log s_{i}^{c}+\gamma \sum_{i=N+1}^{M}\log\left(1-s_{i}^{c}\right)\right\}.$$

The first term sums the classification errors of all predicted points, while the second term sums the classification errors of all candidate points not selected as predictions, representing negative samples. A hyperparameter is used to balance the classification loss weights between positive and negative samples, set to 0.5 in our experiment. The final loss function is

(11) $$L=L_{\mathrm{cls}}+\alpha L_{\mathrm{loc}}.$$

The hyperparameter α balances the two losses and speeds up model convergence.

## 4. Experiments

### 4.1. Experiment Planning

To demonstrate the advantages of our method in crowded and occluded scenarios, we conducted three comparative experiments. We first evaluated our model on the challenging ShanghaiTech PartA and PartB datasets, which represent different crowd densities and scene complexities. Unlike prior works that typically train and test on each dataset independently, we designed a unified evaluation protocol to better assess generalization. Specifically, we first used the model pretrained on PartA to perform inference on PartB, and then trained a unified model on the merged PartA and PartB datasets, and tested it separately on both datasets. This joint training setup allows the model to learn from diverse crowd distributions and scene contexts, thereby improving its robustness. The experimental results validate the effectiveness of our approach across varying levels of crowd density and occlusion, highlighting its superior adaptability and generalization capability.

### 4.2. Datasets Introduction

ShanghaiTech PartA features highly complex scenes with uneven crowd distributions and large variations in scale, making it particularly challenging for detection models. In contrast, PartB contains lower crowd density, relatively uniform scales, and simpler backgrounds, primarily consisting of urban street scenes, with all images sized 786 × 1024. Although PartB is considered less challenging, effective performance still requires the model to generalize well across different contexts and accurately capture critical local features. The diversity between these two subsets highlights the need for strong and adaptive feature extraction capabilities. To address this, our proposed MSCANet integrates an attention-based regression module designed to suppress redundant or irrelevant features while enhancing key spatial and contextual cues. This module enables the network to focus on meaningful crowd-related information and improves prediction consistency, particularly on the simpler yet detail-sensitive PartB dataset. As a result, MSCANet achieves high accuracy and robustness across both subsets, demonstrating its ability to adapt to varied scene complexities and crowd densities.

### 4.3. Implementation Details

In the data preprocessing, each crowd image is normalized and standardized, which can effectively improve the convergence speed of the model. Then, the scaling factor is randomly selected from [0.7, 1.3] for each image to scale to ensure that its short edge is not less than 128. Finally, small images with a fixed size of 128 × 128 are randomly cropped from the original image, and each cropped image is randomly flipped with a 50% probability. In the training phase, we use the Adam algorithm with a fixed learning rate of $1\times 10^{-5}$ to optimize the model parameters, and set the training batch size to 8. The parameter *k* for the truth value attention map is set to 8, and the number of candidate points *M* is set to 1024 to ensure that it is greater than the number of truth points contained in all cropped pictures. At the same time, in Experiment 1, we compared the training of PartA and PartB of ShanghaiTech. In Experiment 2, we compared the fusion trainings of PartA and PartB of ShanghaiTech. In Experiment 3, we predicted PartB with the pretraining model on PartA and cross-verified PartA and PartB with the pretraining model of fusion training.

### 4.4. Result Comparison and Analysis

In order to verify that the number of people predicted by our model in crowded and occluded scenes are closer to the actual count, we conducted a detailed visual comparison with P2PNet, which is one of the leading point-supervised crowd gathering detection methods. Since both approaches rely on point-level supervision, this comparison provides a fair and meaningful assessment of their respective strengths. The visual results demonstrate that our model not only estimates crowd counts more accurately but also better reflects the true spatial distribution of individuals, especially in challenging dense and heavily occluded areas. Furthermore, to comprehensively evaluate the effectiveness of our proposed model, we carried out training and testing on the ShanghaiTech PartA and PartB datasets separately, as well as on a merged dataset that combines both subsets to simulate more complex real-world scenarios. We adopted Mean Absolute Error (MAE) and Mean Squared Error (MSE) as the evaluation metrics, and their calculation methods are as follows:

(12) $$\mathrm{MAE}=\frac{1}{N}\sum_{i=1}^{N}\left|y_{i}^{\prime }-y_{i}\right|,$$

(13) $$\mathrm{MSE}=\frac{1}{N}\sum_{i=1}^{N}{\left(y_{i}^{\prime }-y_{i}\right)}^{2},$$

where $y_{i}^{\prime }$ is the number of the i-th samples predicted by the model, and $y_{i}$ is the real number of the i-th samples. The experimental results show that our model has a very competitive effect.

The results of our model on the ShanghaiTech PartA and PartB datasets are summarized in Table 1. As shown, our model achieves the best performance on PartA, with both MAE and MSE being the lowest values. Compared with the second-lowest results of P2PNet, MAE and MSE are reduced by 1.1 and 3.4, corresponding to reductions of 2% and 3.9%, respectively. On PartB, our model achieves the best MAE, equal to GauNet, while MSE reaches the second-lowest value. Overall, the evaluation demonstrates the superiority of our model.

**Figure 5:**
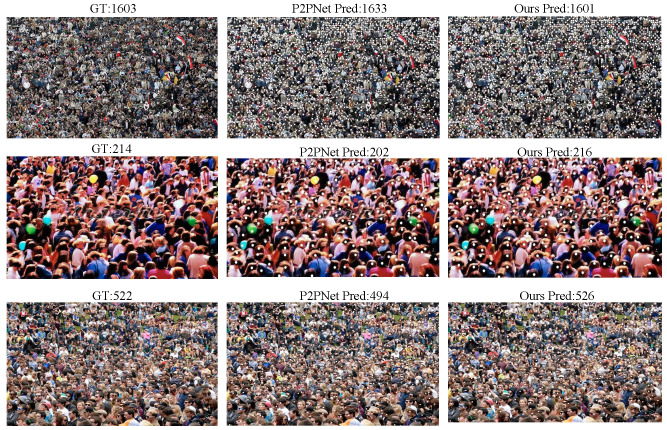
Visual comparison between P2PNet and our model.

To train a more general point-supervised crowd gathering detection model, we also fused PartA and PartB for training and conducted comparisons with two density map methods, as shown in Table 2. Additionally, we performed a visual comparison between our model and P2PNet, as shown in Figure 5, which further illustrates the improvements in feature prediction and localization achieved by our approach.

**Figure 6:**
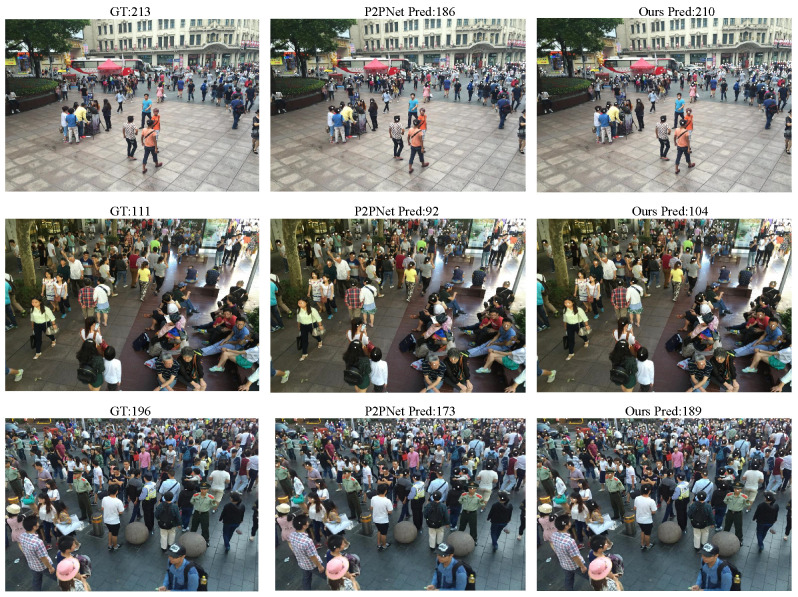
Visual comparison between P2PNet and our model in cross-validation of ShanghaiTech A to B.

**Figure 7:**
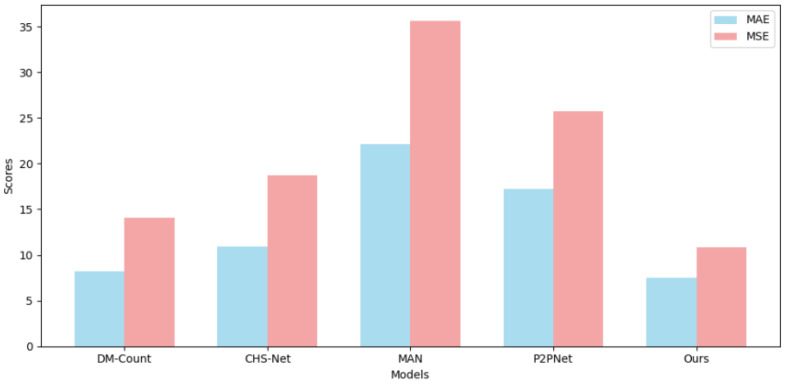
Visual comparison of performance metrics among different models in cross-validation on ShanghaiTech AB to B.

**Figure 8:**
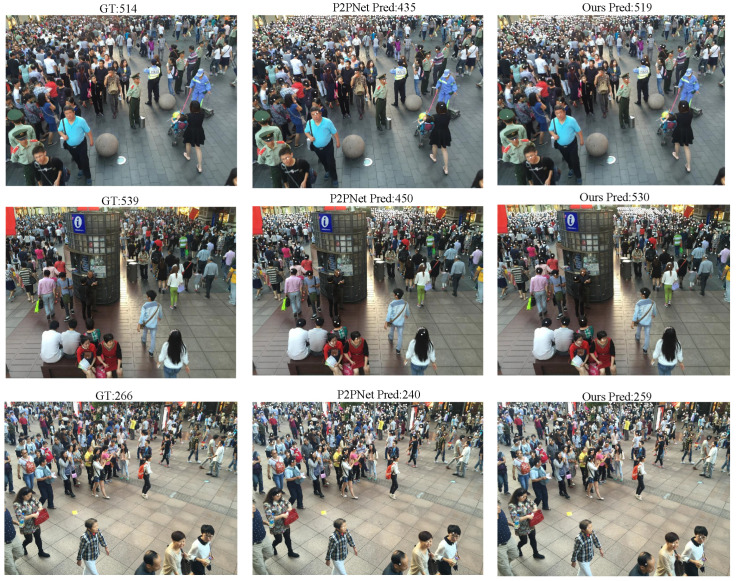
Visual comparison between P2PNet and our model in cross-validation of Shanghai Tech AB to B.

**Figure 9:**
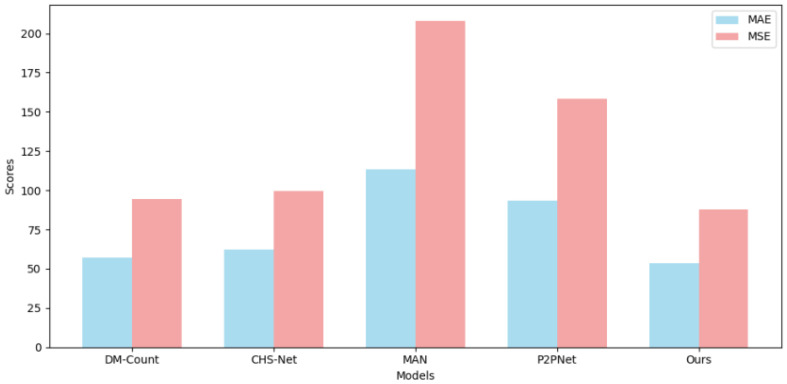
Visual comparison of performance metrics among different models in cross-validation on ShanghaiTech AB to A.

**Figure 10:**
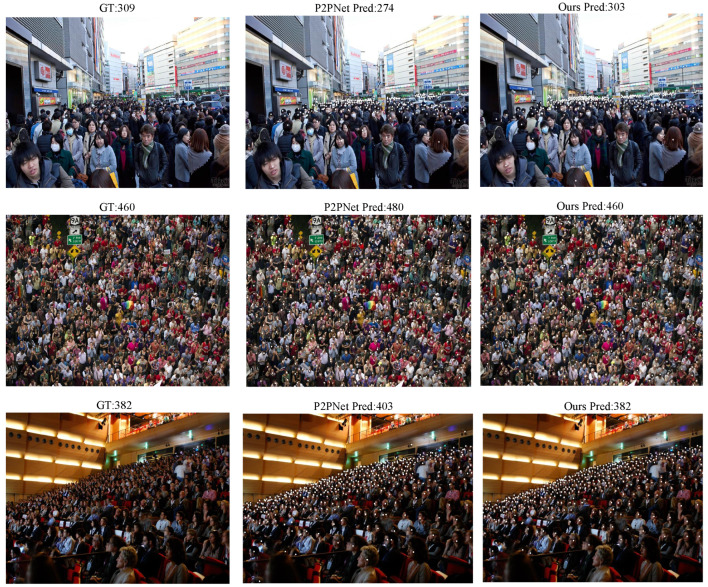
Visual comparison between P2PNet and our model in cross-validation of ShanghaiTech AB to A.

To further evaluate the generalization ability of our model across different datasets, we conducted multiple cross-validation experiments as well as several visualization studies. For example, Table 3 presents the results of predicting the ShanghaiTech PartB dataset using the model pre-trained on ShanghaiTech PartA, with corresponding visual comparisons shown in Figure 6. Table 4 and Figure 7 show the predictions on ShanghaiTech PartB using the model trained on the fused dataset, with additional visual validation provided in Figure 8. Similarly, Table 5 and Figure 9 present the predictions on ShanghaiTech PartA using the model trained on the fused dataset, with corresponding visual verification shown in Figure 10.

We conducted cross-validation experiments on the model across datasets, and the results are as follows: In the cross-validation experiment between PartA and PartB, the MAE and MSE of our model results were 18.2 and 28.7, respectively, which were 3.9 and 5.3 lower than those of the second-ranked P2PNet, that is, 17.6% and 15.5% lower, respectively. In the cross-validation experiment of the AB fusion dataset on PartB, the MAE and MSE of our model results were 7.5 and 10.8, respectively, which were 0.7 and 3.3 lower than those of the second-place P2PNet, that is, 8.5% and 23.4% lower, respectively. In the cross-validation experiment of the AB fusion dataset on PartA, the MAE and MSE of our model results were 53.7 and 87.7 respectively, which were 3.4 and 6.9 lower than that of the second-place P2PNet, that is, 5.9% and 7.2% lower, respectively.

We also selected some visualization results from the cross-validation experiments for comparison. The results show that, in both sparse and dense scenarios, our model consistently outperforms P2PNet in gathering detection. This demonstrates that our model can perform generalized gathering detection across multiple datasets with excellent performance, indicating strong generalization ability and great potential for application in a wider range of real-world scenarios.

### 4.5. Ablation Study

In order to verify the effectiveness of each module we proposed, we conducted ablation experiments on the ShanghaiTech PartA dataset. First, we removed the feature fusion module and directly used the feature map extracted by the backbone network for training. Then, we first compared the different numbers of AGSA modules in the regression branch and the location branch, and then we conducted a comparative experiment on the parameters of the balance location loss function and the confidence score loss function in the loss function. Table 6 shows the influence of different numbers of AGSA modules on the experimental results, demonstrating the effectiveness of each component of the proposed model, and Table 7 validates the rationality of α parameter settings.

The experiments demonstrate that using three AGSA modules in both the positioning and regression branches yields the best performance. Fewer modules reduce effectiveness, while more increase model complexity and hinder training efficiency. Thus, three is a balanced choice. For the learning rate, 0.0002 offers the best trade-off: although 0.0008 slightly lowers MAE to 50.6, it raises MSE to 83.5, indicating poorer performance. Overall, 0.0002 achieves better results in both MAE and MSE.

To validate the use of dilated convolutions in the AGSA module, we conducted ablation experiments by replacing them with depthwise separable convolutions. To match the receptive fields of dilated convolutions with dilation rates of 1, 2, 3, and 4, we used depthwise separable convolutions with kernel sizes of 3, 5, 7, and 9, respectively, and applied appropriate padding to preserve spatial resolution. While the separable convolutions reduce parameters and improve efficiency, experimental results show that dilated convolutions perform better in capturing multi-scale features. The comparison results obtained on the ShanghaiTech Part A dataset are shown in Table 8:

To evaluate the individual contributions of the channel and spatial attention modules within the attention enhancement module, we conducted ablation experiments by separately removing each component, as shown in Table 9.

As shown above, the results highlight the significant impact of incorporating both channel and spatial attention modules. When the channel attention module is removed, the model performance (in terms of MAE and MSE) slightly improves to 53.4 and 91.4, respectively, compared with the baseline. However, when the spatial attention module is removed, the model performance worsens, with MAE reaching 56.7 and MSE rising to 91.3. The best performance is achieved when both channel and spatial attention modules are used together, with MAE improving to 51.6 and MSE reducing to 82.7. This demonstrates that the combination of both modules is crucial for enhancing feature representation, as neither module alone can achieve the optimal performance seen when both are present.

## 5. Conclusions

In this work, we proposed MSCANet, which effectively improves the poor detection effect of the original part of the work in the case of crowding and occlusion by using the convolution attention mechanism and the attention regression branch based on the multi-scale feature fusion. The model has achieved excellent results in multiple public datasets and the fusion datasets we have made. The results show the effectiveness of our work. The model can be used for tasks such as group aggregation event detection in complex scenes, and it is of great help to tasks related to monitoring scenes in the future.

## Figures and Tables

**Table 1:** Performance comparison of the model on ShanghaiTech A and B.

Method	STA		STB	
	MAE	MSE	MAE	MSE
CSRNet [16]	68.2	115.0	10.6	16.0
CLPNet [27]	71.5	108.7	12.2	20.0
AMSNet [28]	56.7	93.4	6.7	10.2
CAN [22]	62.3	100.0	7.8	12.2
S-DCNet [29]	58.3	95.0	6.7	10.7
SGANet [4]	57.6	101.1	6.6	10.2
GauNet [2]	54.8	89.1	6.2	9.9
P2PNet [10]	52.7	85.1	6.3	9.9
DM-Count [30]	59.7	95.7	7.4	11.8
CHS-Net [31]	59.2	97.8	7.1	12.1
TopoCount [29]	61.2	104.6	7.8	13.7
Ours	51.6	82.7	6.2	10.2

**Table 2:** Performance comparison of the model on ShanghaiTech A and B fusion datasets.

Method	MAE	MSE
P2PNet [10]	26.1	58.3
DM-Count [30]	29.7	62.0
CHS-Net [31]	55.5	128.8
MAN [32]	49.4	104.7
Ours	24.4	53.7

**Table 3:** Performance comparison of the model in cross-validation of ShanghaiTech A to B.

Method	MAE	MSE
P2PNet [10]	22.1	34.0
DM-Count [30]	23.1	34.9
CHS-Net [31]	24.6	39.3
MAN [32]	22.1	32.8
Ours	18.2	28.7

**Table 4:** Performance comparison of the model in cross-validation of ShanghaiTech AB to B.

Method	MAE	MSE
P2PNet [10]	8.2	14.1
DM-Count [30]	10.9	18.7
CHS-Net [31]	22.1	35.6
MAN [32]	17.2	25.7
Ours	7.5	10.8

**Table 5:** Performance comparison of the model in cross-validation of ShanghaiTech AB to A.

Method	MAE	MSE
P2PNet [10]	57.1	94.6
DM-Count [30]	62.3	99.5
CHS-Net [31]	113.5	207.8
MAN [32]	93.3	158.3
Ours	53.7	87.7

**Table 6:** Influence of AGSA module number on experimental results.

Number	MAE	MSE
0	53.0	84.9
1	53.5	86.6
2	52.0	83.4
3	51.6	82.7

**Table 7:** Influence of α parameter size on experimental results.

α	MAE	MSE
0.0002	51.6	82.7
0.0004	52.7	85.5
0.0006	52.1	87.6
0.0008	50.6	83.5

**Table 8:** Quantitative comparison of dilated convolution and depthwise separable convolution in the AGSA module.

Method	MAE	MSE
Dilated convolution	51.6	82.7
Depthwise separable convolution	52.7	84.4

**Table 9:** Influence of channel and spatial attention modules on experimental results.

Module	MAE	MSE
Without channel	56.7	91.3
Without spatial	53.4	91.4
With channel and spatial	51.6	82.7

## Data Availability

The ShanghaiTech dataset used in this study is publicly available at https://github.com/desenzhou/ShanghaiTechDataset (accessed on 22 October 2025). Further inquiries can be directed to the corresponding author.
